# Non‐ubiquitous expression of core spliceosomal protein SmB/B′ in chick and mouse embryos

**DOI:** 10.1002/dvdy.537

**Published:** 2022-09-20

**Authors:** Benedict R. H. Turner, Charlotte Mellor, Clara McElroy, Natalie Bowen, Wenjia Gu, Chris Knill, Nobue Itasaki

**Affiliations:** ^1^ Faculty of Health Sciences University of Bristol Bristol UK; ^2^ Faculty of Life Sciences University of Bristol Bristol UK

**Keywords:** cerebro‐costo‐mandibular syndrome, chick, embryo, mouse, SmB/B′, SNRPB

## Abstract

**Background:**

Although splicing is an integral part of the expression of many genes in our body, genetic syndromes with spliceosomal defects affect only specific tissues. To help understand the mechanism, we investigated the expression pattern of a core protein of the major spliceosome, SmB/B′ (Small Nuclear Ribonucleoprotein Polypeptides B/B′), which is encoded by *SNRPB*. Loss‐of‐function mutations of *SNRPB* in humans cause cerebro‐costo‐mandibular syndrome (CCMS) characterized by rib gaps, micrognathia, cleft palate, and scoliosis. Our expression analysis focused on the affected structures as well as non‐affected tissues, using chick and mouse embryos as model animals.

**Results:**

Embryos at young stages (gastrula) showed ubiquitous expression of SmB/B′. However, the level and pattern of expression became tissue‐specific as differentiation proceeded. The regions relating to CCMS phenotypes such as cartilages of ribs and vertebrae and palatal mesenchyme express SmB/B′ in the nucleus sporadically. However, cartilages that are not affected in CCMS also showed similar expressions. Another spliceosomal gene, *SNRNP200*, which mutations cause retinitis pigmentosa, was also prominently expressed in cartilages in addition to the retina.

**Conclusion:**

The expression of SmB/B′ is spatiotemporally regulated during embryogenesis despite the ubiquitous requirement of the spliceosome, however, the expression pattern is not strictly correlated with the phenotype presentation.

## Key Findings

- Spliceosomal protein SmB/B' is expressed in a tissue‐specific manner despite the requirement of splicing in all cells in our body
- In cartilages and palatine mesenchyme cells, SmB/B' is expressed in a sporadic manner: some cells express it strongly while others express it only weakly or do not express it
- Cartilages and tissues that are not affected in CCMS also express SmB/B' prominently, thus the expression is not correlated with the phenotype presentation
- The expression of SmB/B' in the nucleus is correlated to the cell cycle

## 1 INTRODUCTION

The spliceosome is the intranuclear machinery which conducts the splicing of pre‐mRNA into mRNA in a regulated manner. Most genes in metazoans require splicing, among which, more than 99% are processed by the major spliceosome.^1^, ^2^, ^3^ The spliceosome is dynamic in composition, comprising U1, U2, U4, U5, and U6 ribonucleoprotein particles.^4^ Each particle consists of seven core small proteins (Sm) in common (SmB/B′, D1, D2, D3, E, F, and G) and a varying number of other proteins along with small nuclear RNAs forming small nuclear ribonuclear proteins (snRNP).^4^ Each of the particles dynamically assembles in a varying combination throughout the splicing steps together with many splicing co‐factors. Hence, the splicing machinery comprises hundreds of proteins.^1^, ^4^ Because of the universal cellular requirement of the machinery, the core components of the major spliceosome are generally assumed to be expressed ubiquitously across all cell and tissue types.

Despite the essential role of splicing in the body, the phenotype of spliceosomal diseases in humans is highly specific.^5^, ^6^, ^7^, ^8^, ^9^ One example is the cerebro‐costo‐mandibular syndrome (CCMS), a congenital skeletal dysmorphic syndrome caused by loss‐of‐function mutations of *SNRPB* which encodes SmB and its isoform SmB'.^10^, ^11^ The main structures affected in CCMS are the maxilla, mandible, ribs, and vertebra, resulting in cleft palate, micrognathia, rib gaps, and scoliosis, respectively.^12^ These phenotypes are presented in newborn babies and are not progressive after birth, thus, the low level of SmB/B′ expression through *SNRPB* loss‐of‐function mutations seems to affect only limited regions of embryos during the developmental process.

Human cells express two isoforms from the *SNRPB* gene, SmB (231 amino acids) and SmB′ (a.k.a. SmB1, 240 amino acids) by alternative splicing of the seventh (last) exon.^13^, ^14^ SmB and SmB′ differ at the C′‐terminal end in their last 2 and 11 amino acids, respectively. Knock‐down of *SNRPB* is rescued by transfection of either SmB or SmB′ encoding cDNAs, hence, SmB and SmB' are functionally redundant.^14^ While, mice express SmB only (100% amino acid identity to human SmB) and chickens express SmB′ only (240 amino acids, 96% identical to human SmB′).^15^ In this study, these isoforms will be referred to as SmB/B′. Another alternative splicing of *SNRPB* is that the alternative exon 3′ may be included between exons 3 and 4. As the exon 3′ contains a premature termination codon in frame, the resultant protein undergoes nonsense‐mediated decay. Excess amounts of SmB promote the inclusion of exon 3′, suggesting that alternative splicing of exon 3′ works as a part of autoregulation to maintain the expression level of SmB/B′.^10^, ^14^, ^16^

The expression of spliceosomal proteins has been intensively studied, although so far limited to subcellular and subnuclear levels in vitro. Spliceosomal proteins continuously move within the nucleus for their assembly and recycling of the spliceosome.^17^ They may also be found in the cytoplasm during the biogenesis of snRNPs. The small nuclear (sn) RNAs are exported to the cytoplasm, where they bind with target proteins including Sm proteins, thus forming snRNPs, that are imported back into the nucleus.^18^, ^19^, ^20^ During mitosis, Sm proteins diffuse away from the condensed chromatin as the nuclear membrane disassembles, thus, they are passed to daughter nuclei after the cell division together with other snRNPs.^21^, ^22^, ^23^

To help understand the discrepancy between the essential role of the spliceosome and the tissue‐specific congenital phenotype in CCMS, here we investigated the embryonic expression of SmB/B′ using chick and mouse embryos as model animals. We aim to elucidate any correlation between the SmB/B′ expression and the CCMS phenotype. To note, in addition to *SNRPB*, humans and mice also express a paralogue, *SNRPN*, encoding SmN (240 bp) which is a very similar structure to SmB' (93% amino acid matches, 224/240, in both humans and mice).^15^ SmN is expressed mainly in embryonic stem cells, the heart, and the postnatal brain^24^, ^25^ and is functionally interchangeable with SmB/B′.^14^, ^26^ It is well possible that SmN compensates for the reduced expression of SmB/B′ in CCMS patients and thus rescuing the phenotype that would have been presented otherwise. Based on the homology, it is anticipated that SmN in mouse embryos is detected by anti‐SmB/B′ antibodies. Whereas, as chicks do not have *SNRPN*/SmN, the detected expression of SmB' in chick embryos is specific to the expression of *SNRPB*. Because of this, the present study mainly focused on chick embryos, and mouse embryos were used additionally to confirm the conserved expression pattern.

## 2 RESULTS

### 2.1 Expression of SmB/B′ in the neural tube

**FIGURE 1:**
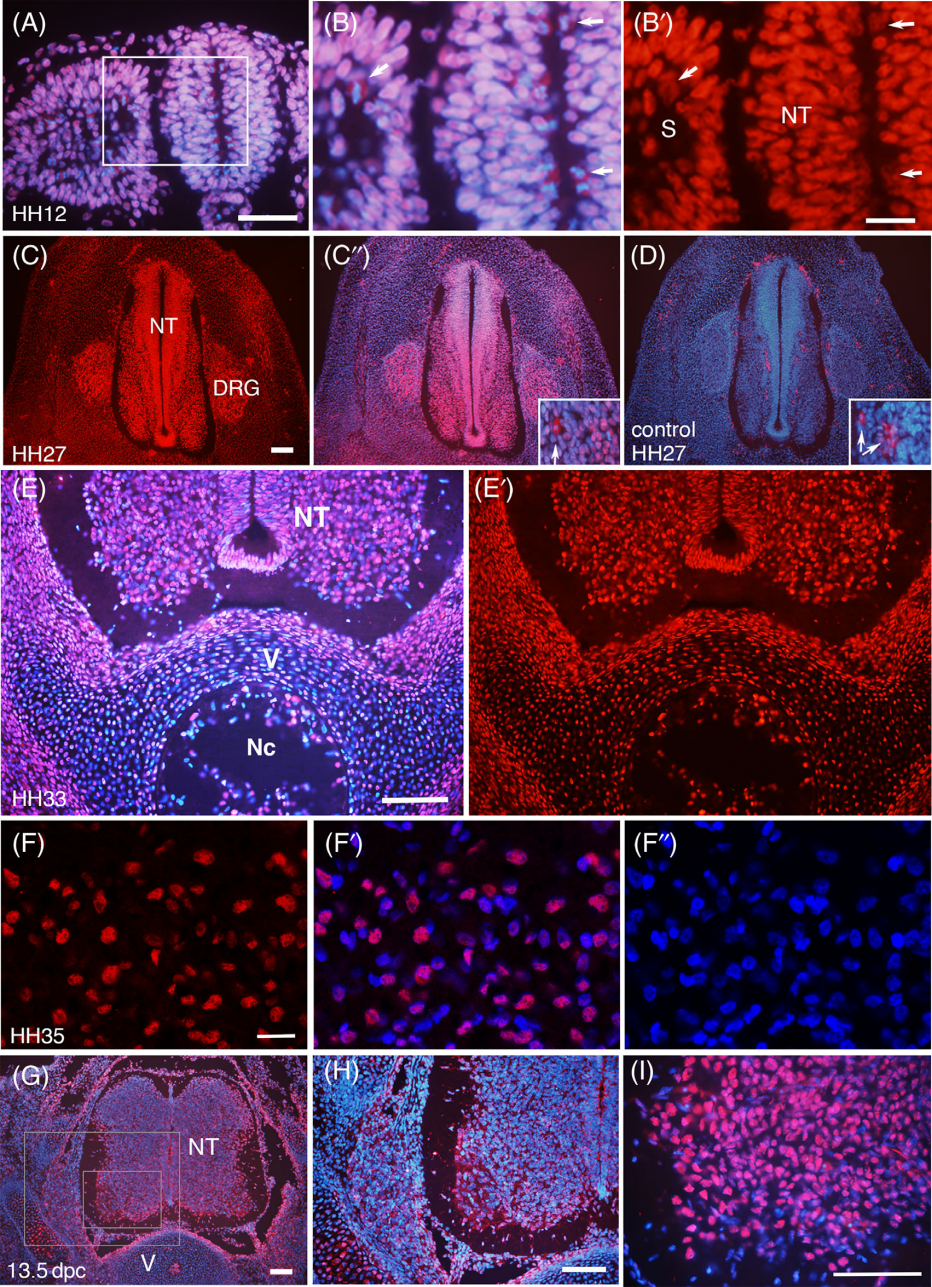
Expression of SmB/B′ in developing neural tube, somite and vertebra. (A‐F) Transverse sections of chick embryos at the trunk level at HH12 (Day 2), HH27 (Day 5), HH33 (Day 8), and HH 35 (Day 9) stained with anti‐SmB/B′ antibody A5 (red) and DAPI (blue), except (D) without the primary antibody. (B), the area magnified as indicated in (A), shows mitotic cells in the ventricular zone of the neural tube (NT) and somitocoele (S) (arrows). (B′) is the single channel showing the anti‐SmB/B′ antibody staining. (C) shows intense staining in the NT and dorsal root ganglion (DRG). The inset in (C′) shows a magnified area at the edge of DRG, where a cell with cytoplasmic staining is seen (arrow), which is considered non‐specific because a similar pattern is seen in (D) without the primary antibody. (E) shows the sporadic expression of SmB/B′ in the ventral column of the NT, vertebra (V), and notochord (Nc). (F‐F″) are confocal images of the ventral column of the NT. (G‐I) Transverse sections of mouse embryos at 13.5 dpc, stained with anti‐SmB/B′ antibody A5 (red) and DAPI (blue). (H) and (I) are magnifications of (G) as indicated by the rectangles. NT, neural tube; V, vertebra. Scale bars in (A) 50 μm; (B and F) 20 μm; (C, E, G‐I) 100 μm

In 2‐day‐old chick embryos at Hamburger Hamilton stage 12 (HH12),^27^ the expression of SmB/B′ was homogeneous and ubiquitous, and localized in the nucleus (Figure 1A,B). Mitotic cells with the condensed chromatin were seen on the ventricular surface of the neural tube and at the center of epithelialized somites, where SmB/B′ staining was spread in the whole cells due to the diffusion of the nucleoplasm during mitosis.^21^

At HH27 (Day 5), cells in the neural tube and dorsal root ganglia (DRG) showed strong staining of SmB/B′, whereas other tissues, such as somite derivatives, showed relatively low staining (Figure 1C). Negative controls without the primary antibody showed non‐specific staining in the cytoplasm (Figure 1D). As we were not able to detect the origin of the non‐specific signal, we ensured in the following analyses that negative controls were accompanied at all times when interpreting the result.

As development proceeds on Days 8‐9 (HH 33‐35), the neural tube, primordial cartilages and notochord showed a sporadic expression of SmB/B′; some cells showed strong expression while other cells in the same tissue were negative or only faintly stained (Figure 1E,F). The sporadic pattern was seen in the ventral column of the neural tube, notochord, and developing vertebrae especially around the notochord, whereas the edge of the vertebra (away from the notochord) showed almost all cells stained positive (Figure 1E,F). This was also the case in mouse embryos (Figure 1G‐I).

### 2.2 Expression of SmB/B′ in ribs and vertebrae

**FIGURE 2:**
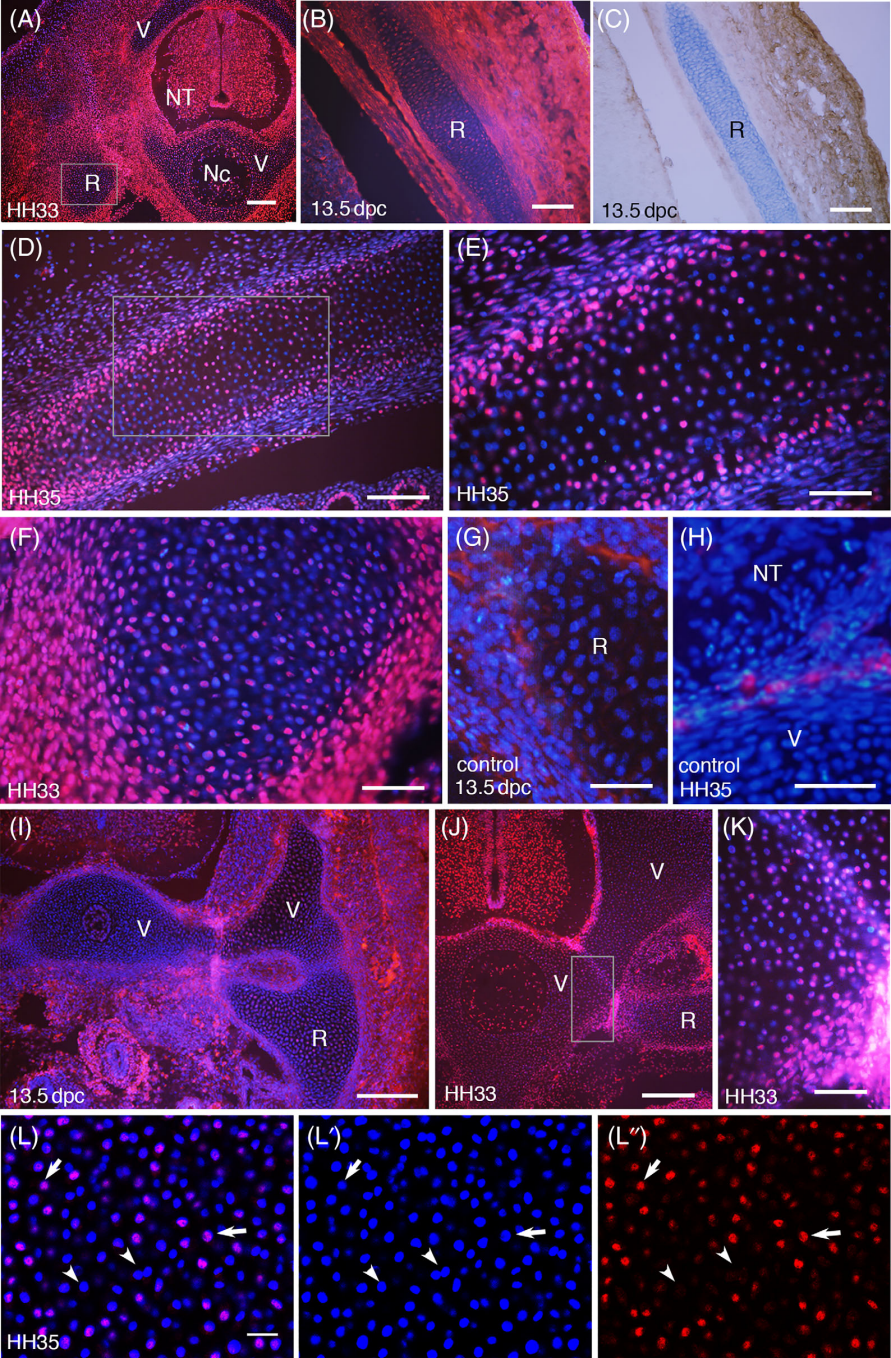
Expression of SmB/B′ in the rib and vertebra. Transverse sections of chick (A, D‐F, H, J‐L) and mouse (B, C, G, I) embryos at indicated stages, stained with anti‐SmB/B′ antibody (red) and DAPI (blue) except (C, G, H). Nc, notochord; NT, neural tube; R, rib; V, vertebra. (A) Montage displaying the large picture, with a rectangle indicating the area magnified in (F). Figure 1E is also a high magnification of (A). (B and C) Thoracic body wall including the rib (R) at adjacent sections. (C) is stained with anti‐SmB/B′ antibody (brown) and Alcian blue (light blue) for cartilage. (D‐F) Magnified view of the rib. (E) and (F) are high magnification of the rectangular part in (D) and (A), respectively. (G and H) Negative controls without primary antibodies. The pericardium of the rib (R) or vertebra (V) in (G and H) show non‐specific cytoplasmic staining. (I‐K) The vertebra (V) and rib (R) forming the costovertebral joint. (K) is a high magnification of the area indicated in (J). (L‐L″) Confocal images of the rib in chick, showing SmB/B′‐positive (arrows) and negative (arrowheads) nuclei. Scale bars in (A, I, J) 200 μm; (B‐D) 100 μm; (E‐H, K) 50 μm, (L) 20 μm

**FIGURE 3:**
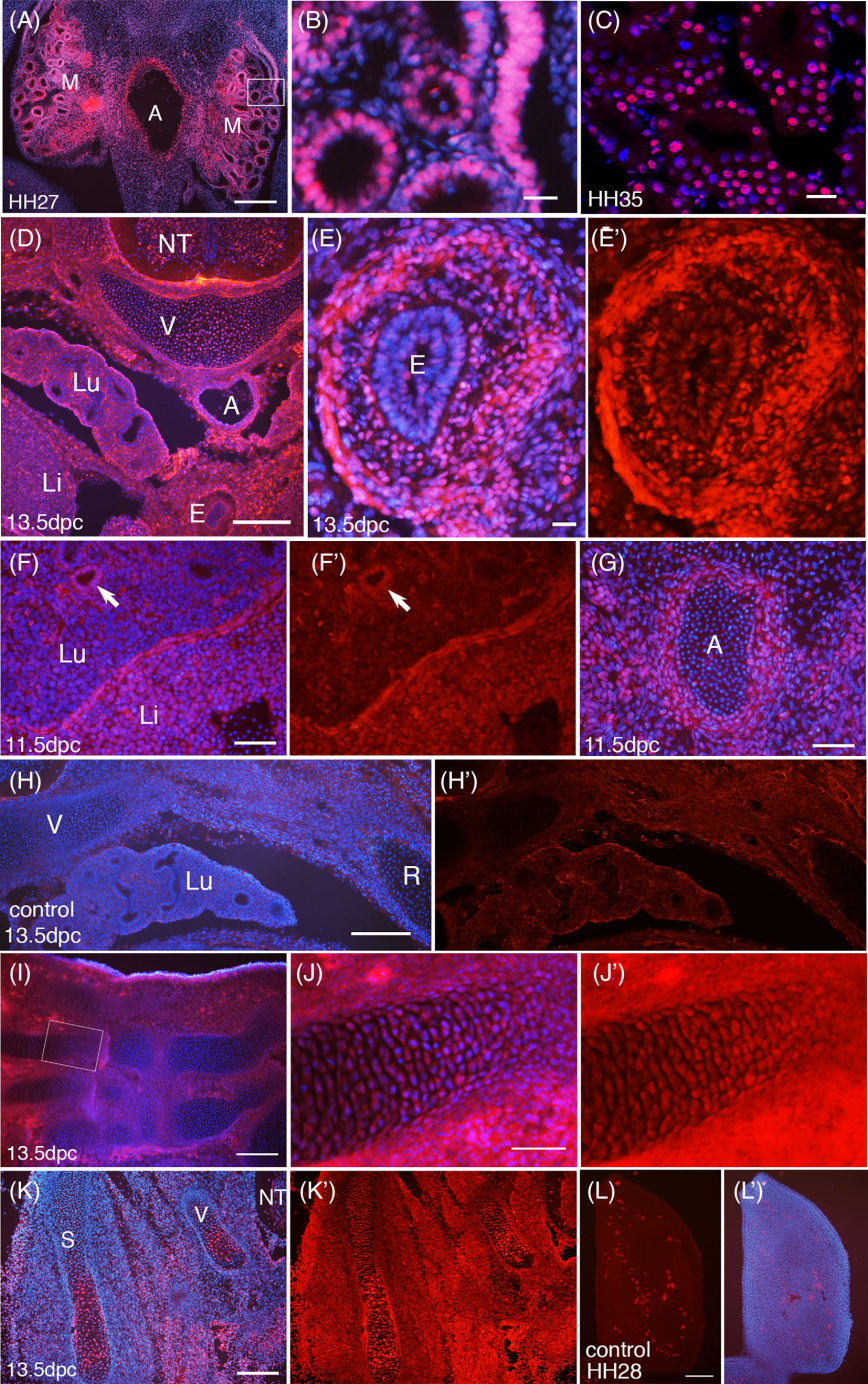
Expression of SmB/B′ in visceral organs and limbs. Sections of chick (A‐C, L) and mouse (D‐J) embryos at indicated stages, stained with anti‐SmB/B′ antibodies (red) and DAPI (blue). A, aorta; E, esophagus; Li, liver; Lu, lung bud; M, mesonephros; NT, neural tube; R, Rib; S, scapula; V, vertebra. (A‐C) Transverse sections of chick embryos with the aorta (A) and mesonephros (M). The epithelia of mesonephric tubules are strongly positive for SmB/B′. (C) is a confocal image of the mesonephros. (D‐H) Transverse sections of mouse embryos at the level of the thorax‐abdomen border, with enlarged views of the esophagus in (E), the lung bud and liver in (F), and the aorta in (G). The arrow in (F) shows the lung epithelium. (H and H′) are non‐specific staining without primary antibodies. (I and J) Palmar plane sections at the carpal area. The limb cartilages show sporadic nuclear staining. (K) The area lateral to the neural tube (NT), including the scapula (S) and the arch of the vertebra (V). In the scapula, the dorsal half (the upper side in the panel) shows nuclei with weak SmB/B′ staining, whereas the ventral half shows strong SmB/B′ staining. The arch of the vertebra also shows strong nuclear staining at the core. On this section, the anti‐SmB/B′ antibody of Y12 clone was used. On all other embryo sections, A5 clone was used. (L) A negative control staining without primary antibody, showing the limb bud with some mesenchymal cells strongly stained in the cytoplasm. Scale bars in (A, D, H, I, K, L) 200 μm; (F, G, J) 50 μm, (B, C, E) 20 μm

Ribs and vertebrae are morphologically structured and chondrogenesis has started by HH33‐35 (Day 8‐9) in chick and 13.5 dpc mouse embryos. Chondrocytes proliferate rapidly by signals from the perichondrium, after which, they arrest the cell cycle, become post‐mitotic hypertrophic chondrocytes and produce a large amount of cartilage matrices.^28^ As a result, the nuclei are sparsely distributed compared to other tissues (Figure 2A) and the cartilage is detectable with Alcian blue staining (Figure 2B,C). Chondrocytes located close to the perichondrium continue receiving signals and proliferating, which helps remodeling in size and shape, whereas the majority of hypertrophic chondrocytes at the core will undergo apoptosis and be replaced by endochondral ossification.^28^ In agreement with this, the section of rib cartilages showed densely packed nuclei at the edge of the cartilage with strong staining of SmB/B′ and sparsely distributed nuclei at the center with speckled staining pattern (Figure 2D‐F). Negative controls without the primary antibody showed cytoplasmic staining in the perichondrium (Figure 2G,H). Because of this, the strong staining at the costovertebral joint was suspected to include non‐specific staining (Figure 2I‐K). Nonetheless, specific nuclear staining was seen strongly and homogeneously near the joint, while the speckled pattern with sporadically negative nuclei is seen away from the joint (Figure 2I‐L). The sporadic staining in developing cartilages was seen not only in ribs and vertebrae (Figures 1E and 2) but also in the limb and scapula (see Figure 3).

### 2.3 Expression of SmB/B′ in visceral organs and limbs

The most prominent staining of SmB/B′ in visceral organs was in the mesonephros, where the tubular epithelium displayed strong nuclear staining, while not as strong in the underlying mesenchymal cells (Figure 3A‐C). The esophagus, on the contrary, showed only weak nuclear staining in the epithelium while the underlying mesenchyme was strongly stained (Figure 3D,E). Developing lungs showed relatively weak staining compared to the liver, except for the lung epithelium (Figure 3F). The wall of the aorta was positive in both nuclei and the cytoplasm, whereas the blood cells were negative (Figure 3G). Negative control staining revealed non‐specific signals in the basal lamina and a background level of staining in mesenchymal cells (Figure 3H). Limb cartilages showed sporadic nuclear staining (Figure 3I,J) similar to vertebrae and ribs (Figure 2). In the mouse developing scapula, the ventral side of the core part showed sporadic yet strong staining in the nucleus, whereas the dorsal half showed relatively weak staining at 13.5 dpc (Figure 3K). This likely reflects the development of the scapula which is formed from the ventral part (the future humerus joint part),^29^, ^30^ hence, the ventral side is more advanced than the dorsal side in differentiation. To note, in Figure 3K,K′, a different clone of SmB/B′ antibodies, Y12, was used, while in other panels A5 clone was used, to confirm the sporadic expression pattern in the cartilage. The negative control of the limb bud demonstrated that a small number of cells show strong cytoplasmic signals in the mesenchyme (Figure 3L).

### 2.4 Expression of SmB/B′ in the head region

**FIGURE 4:**
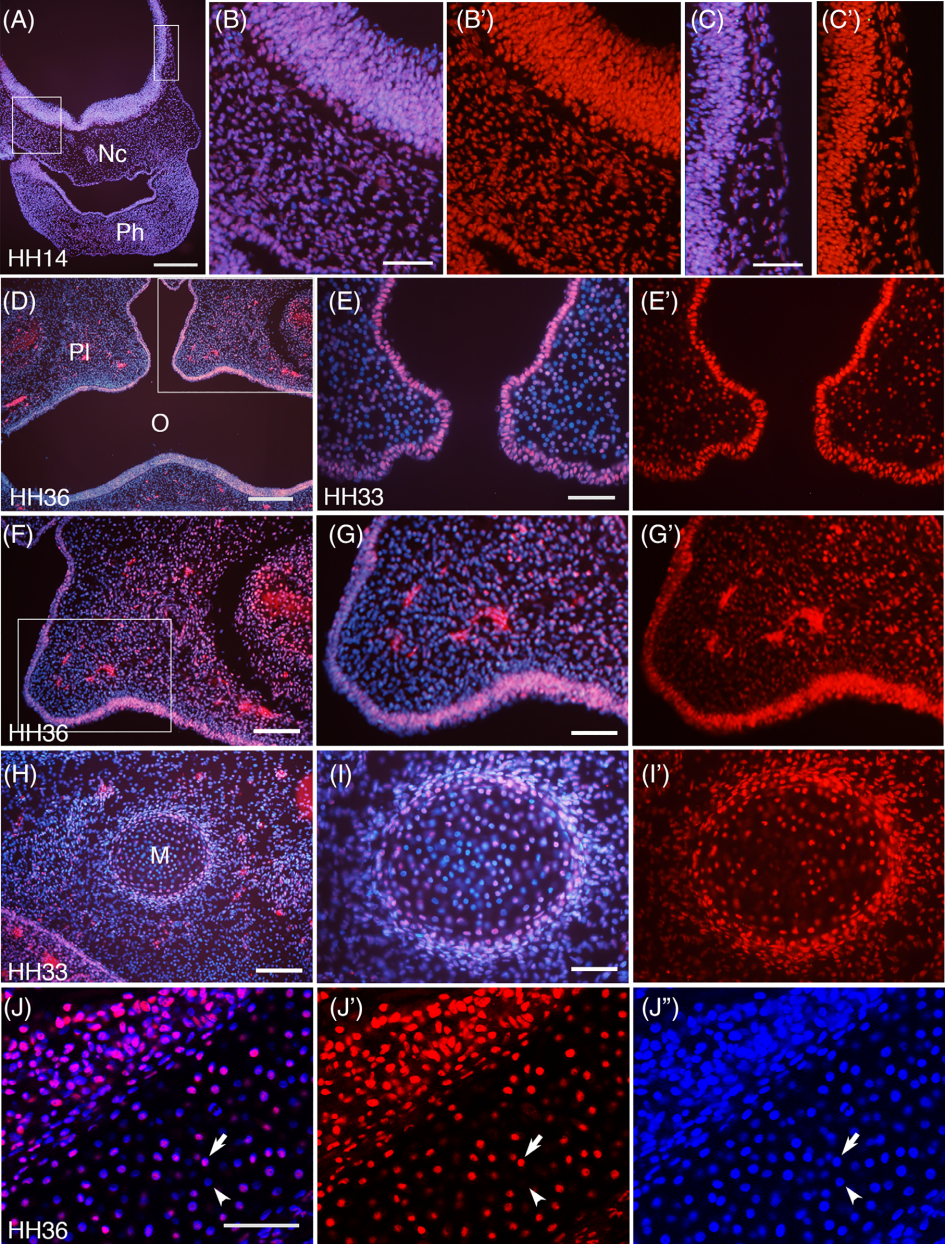
Expression of SmB/B′ in the head, maxilla, and mandible. Frontal sections of chick embryos at indicated stages, stained with anti‐SmB/B′ antibody A5 (red) and DAPI (blue). Nc, notochord; Ph, first pharyngeal arch; Pl, palate; O, oral cavity; M, Meckel's cartilage. (A) The hindbrain with fused first pharyngeal arch (Ph), with rectangles indicating the area magnified in (B) and (C). Ubiquitous staining in both neuroepithelium and underlying cells. (D‐G) The palate region. (D) indicates the magnified area of (F), and (F) indicates the area magnified in (G). (E) is at a younger stage than (D, F, G), showing sporadic expression of SmB/B′ in the mesenchymal cells. The strong staining seen in the HH36 mesenchyme is considered non‐specific, as seen in Figure 3L. (H‐J) The Meckel's cartilage with a sporadic expression of SmB/B′ at the core part and strongly‐stained chondrocytes and perichondrium at the peripheral part. (J) is a confocal image with the perichondrial part at the upper‐left side of the panel and the core part of the cartilage at the lower‐right side of the panel with SmB/B′‐positive (arrow) and negative (arrowhead) nuclei. Scale bars in (A and D) 200 μm; (B, C, E, G, I, J) 50 μm; (F and H) 100 μm

In chick embryos at HH14 (Day 3), the head neural tube as well the mesenchymal cells, including neural crest cells and mesodermal cells, were all homogeneously stained for SmB/B′ (Figure 4A‐C). At HH 33‐36 (Days 8‐10), bilateral palatal shelves come close at the midline as a process of palatine formation, where the overlying epithelium is strongly positive (Figure 4D,E). The underlying mesenchyme, to the contrary, showed sporadic expression at HH33 (Figure 4E), which later became more ubiquitous and suppressed on the medial side (Figure 4D,F,G). In the mandibular arch, the most prominent structure was Meckel's cartilage, which showed a sporadic expression of SmB/B′ in the core part of the cartilage and strong and homogeneous expression in the peripheral part of the cartilage and the perichondrium (Figure 4H‐J), similarly to the ribs and vertebra (Figures 1E and 2D‐F).

### 2.5 Expression of SmB/B′ in the eye

**FIGURE 5:**
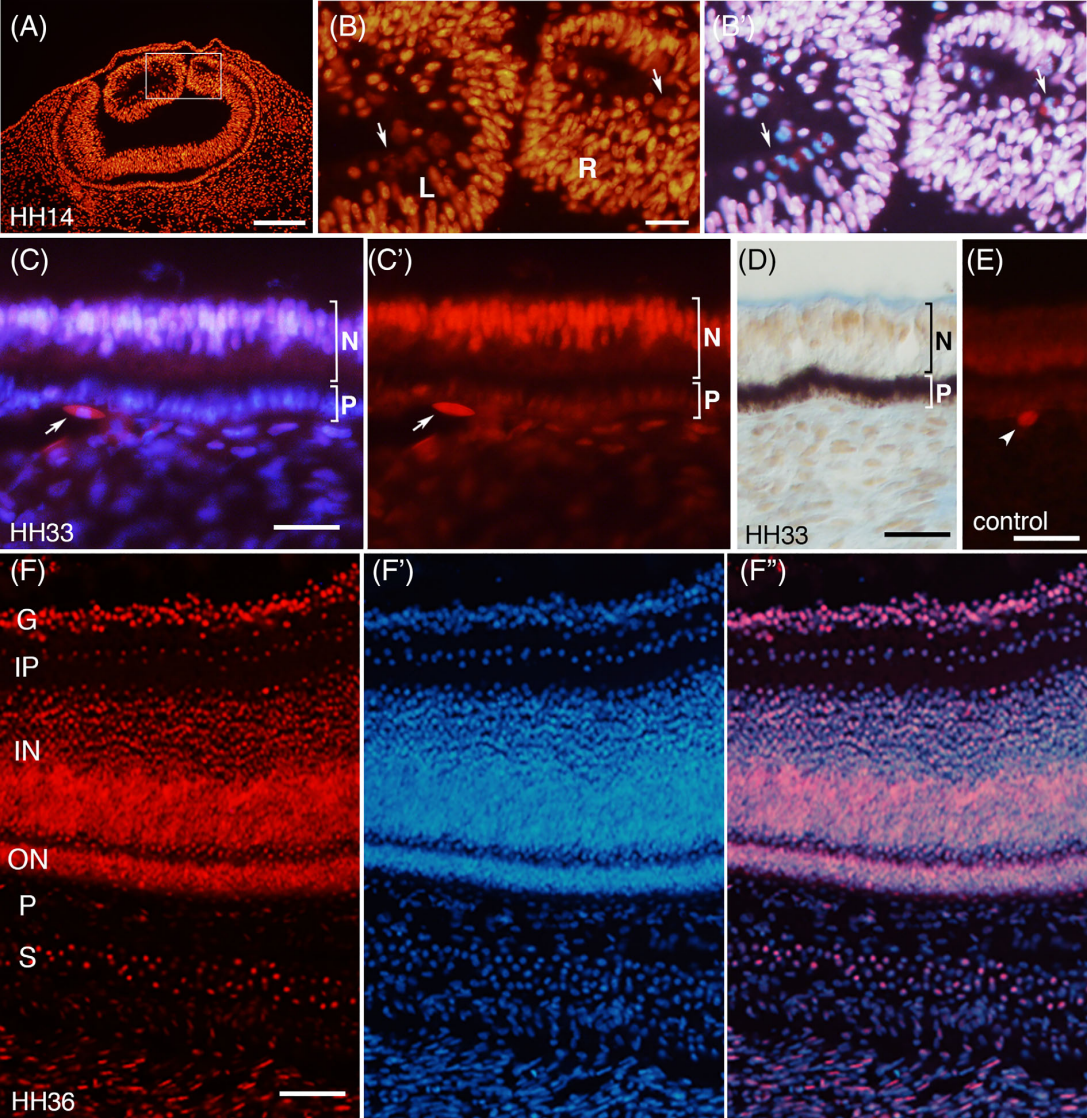
Expression of SmB/B′ in the eye. Transverse sections of developing eyes in chick embryos at indicated stages, stained with anti‐SmB/B′ antibody A5 (red) and DAPI (blue), except that in (D) the antibody was visualized in brown (Alcian blue staining left non‐specific blue color at the vitreous surface) and in (E) the primary antibody was not used. (A and B) The ubiquitous and strong expression at HH14, in the lens (L), the retinal epithelium (R) and head mesenchyme. Mitotic cells (arrows) are seen at the center of the lens and retinal epithelium. The area shown in (B) is indicated as a rectangle in (A). (C‐E) At HH33, the retinal neuroepithelium (N) shows strong nuclear staining compared to the pigment cell layer (P) and underlying periocular mesenchyme. The dark color in the pigment epithelium in (D) is the endogenous pigmentation of the cells. Arrow in (C) indicates a mesenchyme cell with cytoplasmic staining which is likely non‐specific, as similar staining is also seen in the negative control (E, arrowhead). (F) At HH36, the neuroretina has differentiated into the ganglion cell layer (G), inner plexiform layer (IP), inner nuclear layer (IN) and outer nuclear layer (ON), in which SmB/B′ is expressed in most of the cells except the IP layer which shows weak expression. Underneath is pigment cell layer (P) which is negative for SmB/B′ and developing choroid and scleral cartilage (S) which shows sporadic and nuclear‐localized staining. Some of the underneath mesenchyme cells show staining in the cytoplasm. Scale bars in (A) 100 μm; (B, C, F) 20 μm

The eye primordium at HH14 (Day 3) of chick embryos showed strong staining of SmB/B′ in both lens and retinal epithelium (Figure 5A,B). Similar to the neuroepithelium in Figure 1, mitotic cells were present in the retinal epithelium at the surface originally facing the ventricle, as well as at the center of the lens (Figure 5B). At HH33 (Day 8), the neuroretinal layer showed strong nuclear staining compared to the pigment cell layer and underlying periocular mesenchyme (Figure 5C,D). In the mesenchyme, some cells showed cytoplasmic staining (Figure 5C) which was also seen in the negative control like other regions (Figure 5E), hence deemed non‐specific. At HH36 (Day 10), the neuroretina expressed SmB/B′ strongly in the ganglion cell layer and inner and outer nuclear layers, whereas only weak expression was detected in the inner plexiform layer and pigment cell layer (Figure 5F). The scleral cartilage showed the distinct sporadic nuclear staining (Figure 5F).^31^

### 2.6 Expression of *SNRPB* and *SNRNP200* detected by RNA in situ hybridization

**FIGURE 6:**
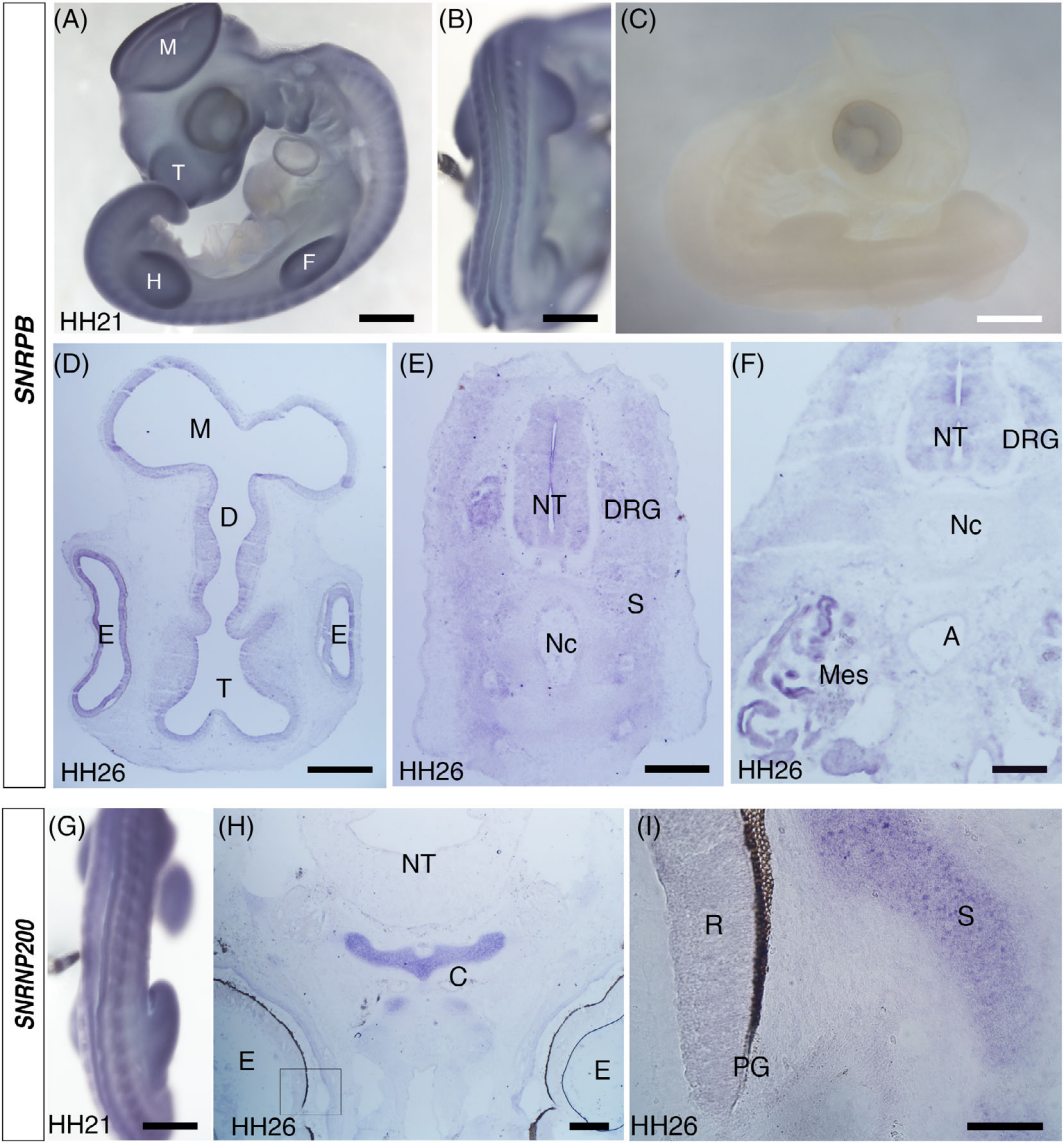
RNA in situ hybridization of *SNRPB* and *SNRNP200* on chick embryos. (A‐C) Whole‐mount staining of chick embryos with *SNRPB* probe at HH21. The bulging structures, the telencephalon (T), mesencephalon (M), forelimb (F), and hindlimb (H) are labeled. In the dorsal view (B), the periodic pattern of somites is seen. (C) is an embryo hybridized with a sense probe as a negative control. (D‐F) Horizontal sections of chick embryos at HH26, hybridized with *SNRPB* probe. A, aorta; D, diencephalon; DRG, dorsal root ganglion; E, eye; M, mesencephalon; Mes, mesonephros; Nc, notochord; NT, neural tube; S, sclerotome; T, telencephalon. (G) Whole‐mount staining with *SNRNP200* probe at HH21, the dorsal view. The periodic pattern of somites is similar to (B). (H and I) A horizontal section at HH26 at the head/neck level, hybridized with *SNRNP200* probe. The neural tube of the hindbrain (NT), the eye (E), and developing cartilage (C) of the skull base are seen. The area magnified in (I) is shown as a rectangle. In (I), the retina (R), pigment cell layer (PG), and scleral cartilage (S) are seen. Scale bars in (A‐C and G) 1 mm; (D‐F) 200 μm; (H) 500 μm; (I) 100 μm

In wholemount specimens of chick embryos, RNA in situ hybridization of *SNRPB* showed broad staining with a specific pattern in somites (Figure 6A‐C). Hybridization on sections revealed that the expression in the head is localized to the neuroepithelium and retina (Figure 6D). At the trunk level, the most prominently stained structures were the neural tube, DRG, sclerotome cells surrounding the notochord and mesonephros (Figure 6E,F). The patterns complement the immunohistochemical results, including the expression in the neural tube and DRG as seen in Figure 1C and mesonephros in Figure 3A. The aorta, which showed both nuclear and cytoplasmic staining in Figure 3G, was presented with a low level of expression (Figure 6F).

So far, our results have demonstrated that *SNRPB*/SmB/B′ is expressed in developing cartilages of not only the ribs and vertebrae where CCMS patients present the phenotype but also in other cartilages, as well as other tissues such as the neural tube, retina, and visceral organs that are not affected in CCMS patients, yet the expression is not ubiquitous. Thus, it appears that the non‐ubiquitous expression pattern does not strictly correlate with the tissues and organs that are affected in CCMS patients. To explore this further, we examined the expression of another snRNP gene, *SNRNP200*, which encodes a component of the U5 particle. Mis‐sense mutations of the gene cause retinitis pigmentosa without affecting skeletal development.^32^, ^33^, ^34^ Whole mount staining of chick embryos showed broad staining with periodic staining in somites, similar to the pattern of *SNRPB* expression (Figure 6G). In the head region, developing cartilages of the skull base and sclera expressed *SNRNP200* strongly compared to the surrounding tissues (Figure 6H,I). The expression of *SNRNP200* in the neuroretina (retina excluding the pigment cell layer) was not as strong as the scleral cartilage (Figure 6I). The result is reminiscent of that of *SNRPB*/SmB/B′ expression in the sense that the overall expression is broad, yet some tissues are specifically strong, and the strong expression is not necessarily limited to the tissues that are affected by the mutation in human.

### 2.7 Expression of SmB/B′ detected by Western blotting

**FIGURE 7:**
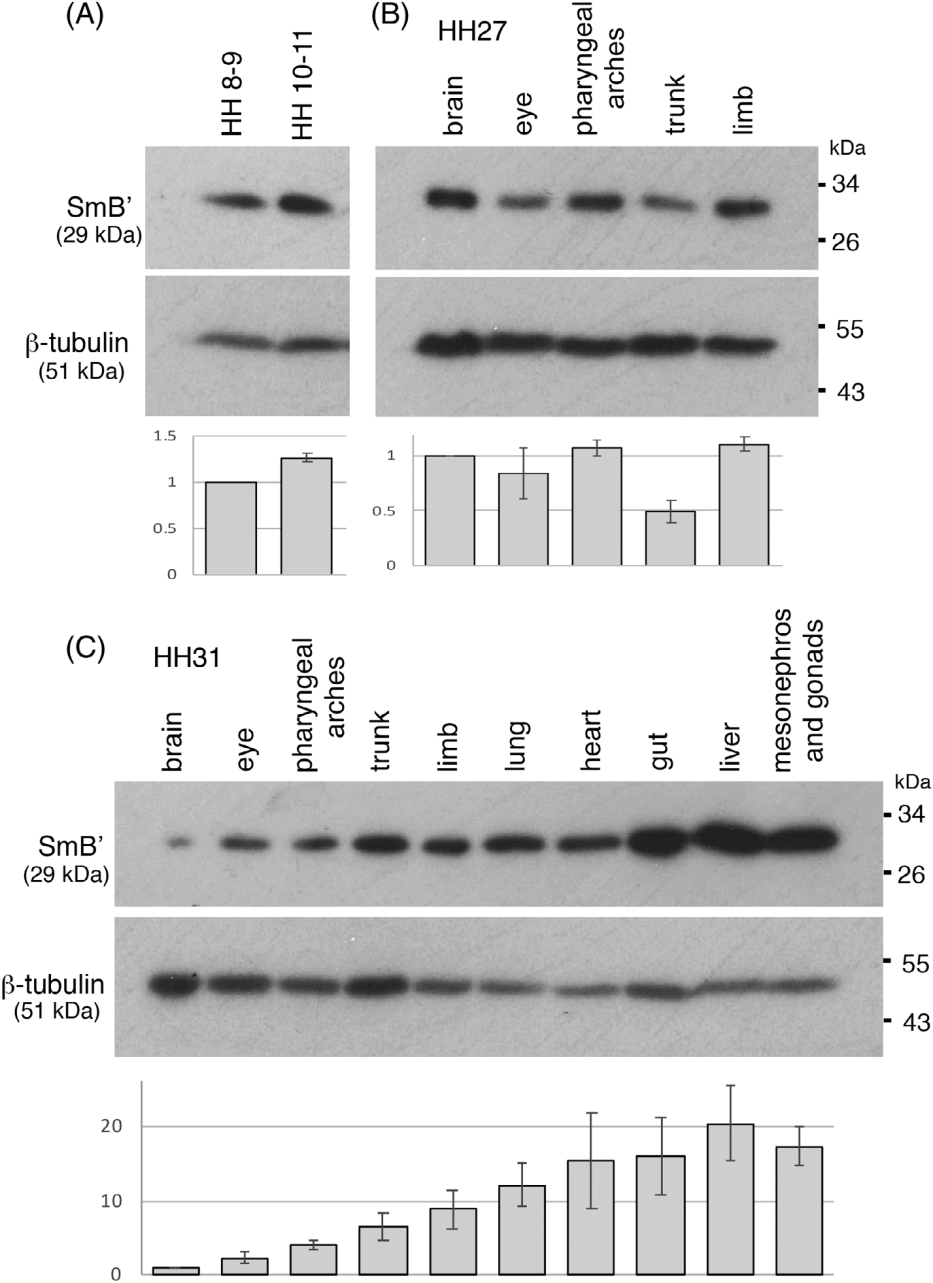
Western blot analysis of SmB' in chick embryos. Western blots of chick embryo extract on Day 2 at HH 8‐9 and HH 10‐11 (A), Day 5 at HH27 (B), and Day 7 at HH31 (C). β‐tubulin is a loading control. Below bar graphs show quantification of band intensity of SmB' normalized to that of β‐tubulin, and further normalized to the first lane's samples. Data shows the average ± Std of two independent loadings with two independent exposures (n = 4).

The expression of SmB', the only isoform in chick encoded by *SNRPB*, was examined on Western blots at four different stages; HH 8‐9 and HH 10‐11 stages on Day 2 in whole embryos and at HH 27 (Day 5) and HH 31 (Day 7) by dissecting various regions (Figure 7). Single bands were detected at the anticipated size of 28 kDa. In the comparison between different regions of tissues at HH27, expression in the trunk was relatively low compared to the brain, eye, pharyngeal arches, and limbs (Figure 7B). This appeared against the results obtained from the immunohistochemical studies in Figures 1 and 3, where strong and specific expression was seen in the neural tube and mesonephros in the chick trunk. This may be because the specific staining highlights a relatively small population of SmB' expressing cells. As such, the quantitative aspect of Western blot results is for guidance only. At HH31, which is developmentally equivalent to mouse 13.5 dpc, the embryos were dissected into different organs (Figure 7C). The brain appeared to express SmB' at a relatively low level, whereas visceral organs tend to express it at higher levels by comparison. Overall, all organs/body parts of chick embryos examined in this study expressed SmB'.

### 2.8 Expression of SmB/B′ in cell lines

**FIGURE 8:**
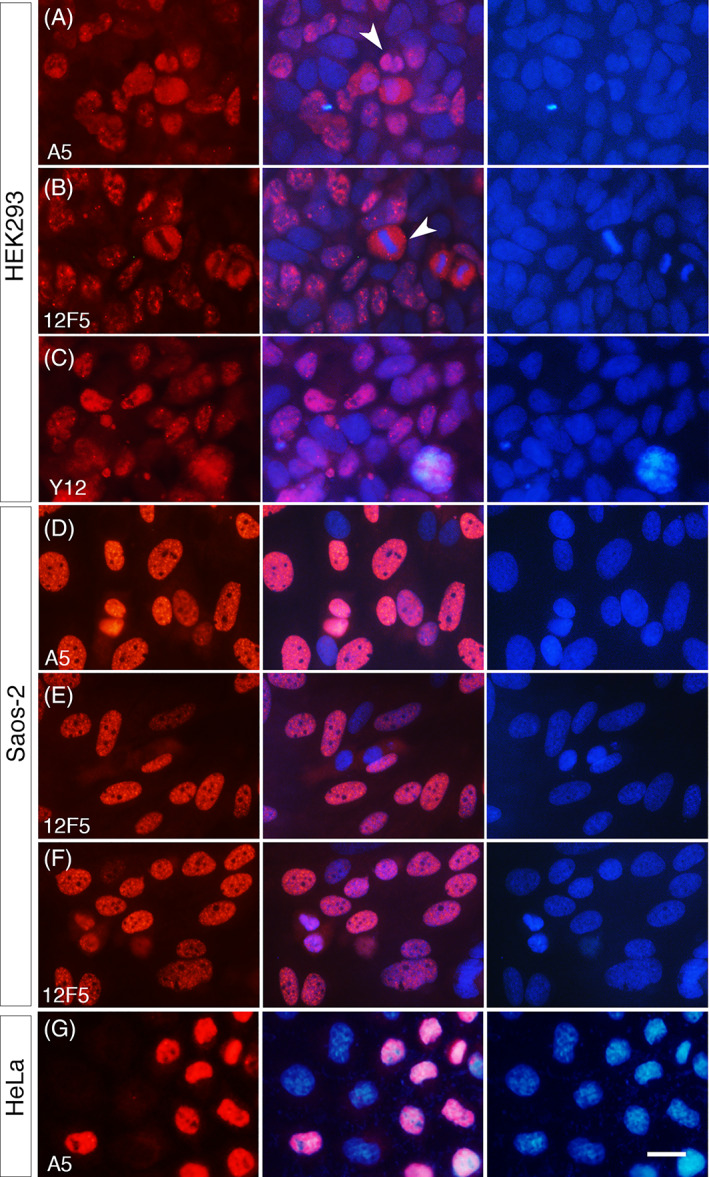
Expression of SmB/B′ in cell lines. HEK293 (A‐C), Saos‐2 (D‐F), and HeLa (G) cells were stained with indicated SmB/B′ antibodies (A5, 12F5, or Y12). Arrowheads in (A and B) indicate cells at the metaphase with a clear chromatin alignment. Scale bar, 20 μm for all panels

The sporadic expression of SmB/B′ in cartilages and the ventral neural tube (Figures 1, 2, 3, 4, 5) led us to investigate the expression in homogeneous cell populations in cultured cell lines. HEK293, Saos‐2 and HeLa cells were tested with three different SmB/B′ antibodies. HEK293 was used based on the previous study that one of the prominent CCMS phenotypes, proximal rib defects, was phenocopied by modulating the Wnt and BMP pathways^35^ and HEK293 cells are responsive to both pathways.^36^ Saos‐2 cells were used because of the osteoprogenitor‐like feature,^37^ which was considered relevant to the skeletal phenotype presented in CCMS patients. HeLa cells were used because the intensive transcriptome analysis of *SNRPB* knock‐down was conducted using the cell line by Saltzman et al.^14^ These cell lines, all derived from human tissues, also allowed us to examine the expression of SmB/B′ in human cells. All cell lines showed nuclear‐specific staining, however, in a mixed intensity; some cells were weakly stained compared to others (Figure 8). Cells at the M phase with condensed chromatin showed strong staining throughout the whole cell (Figure 8A,B), consistent with the in vivo results (Figures 1B and 5) and other reports.^21^, ^22^, ^23^

**FIGURE 9:**
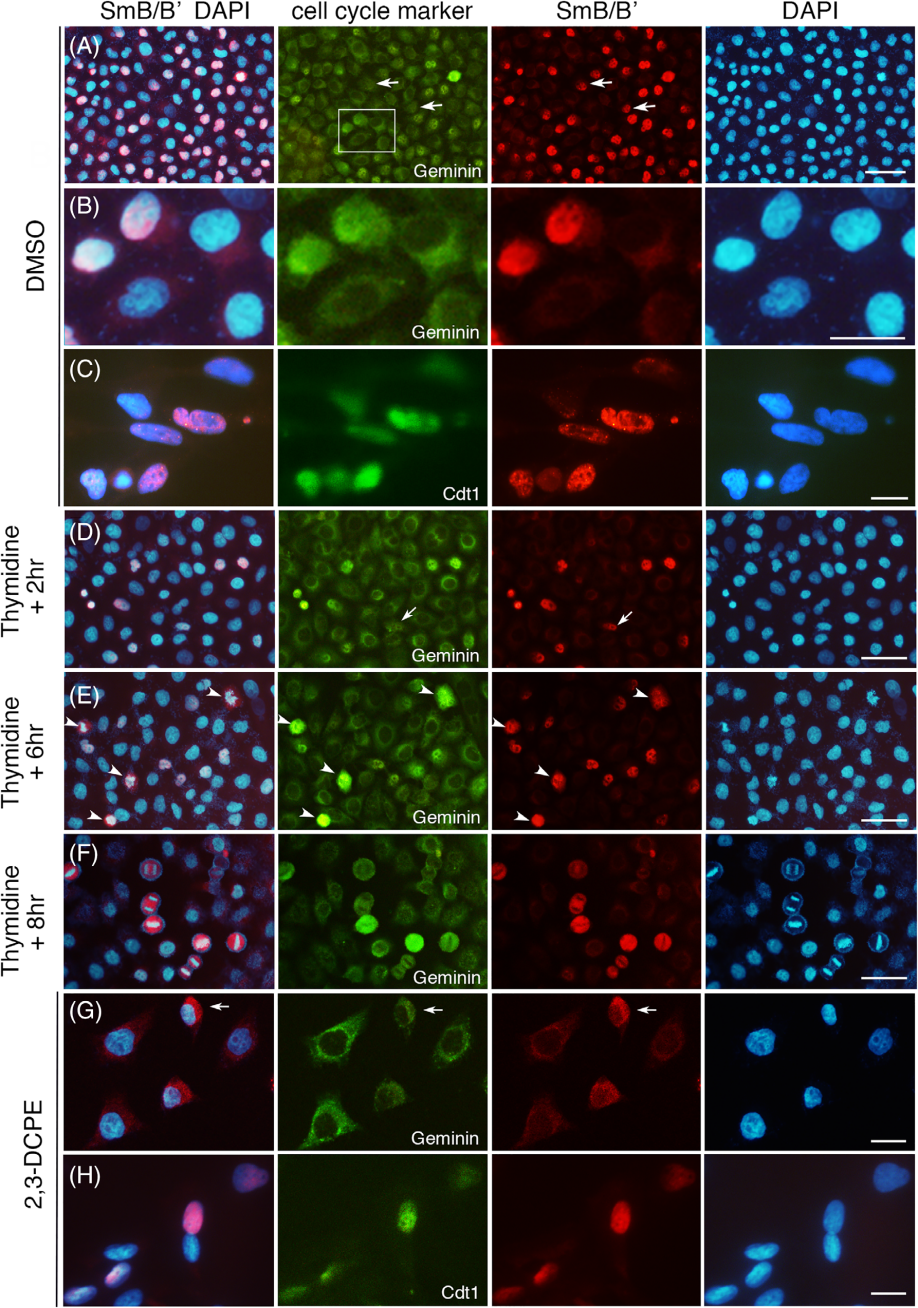
Expression of SmB/B′ in HeLa cells after double‐thymidine treatment. HeLa cells were treated with either DMSO (control, A‐C), thymidine (D‐F) followed by further incubation for cell recovery for indicated hours or 2,3‐DCPE (G and H) and stained with anti‐SmB/B′ (clone A5, red), anti‐Geminin (green) or anti‐Cdt1 (green) antibodies and DAPI (blue). The left column is the overlay of SmB/B′ and DAPI. In (A), SmB/B′‐positive cells with indistinct nuclear localization of Geminin are indicated by arrows. In the Geminin panel of (A), the area magnified in (B) is indicated by a rectangle. (B) shows that SmB/B′ staining is very faint or negative in the cells which express Geminin in the perinuclear region. (C) shows co‐expression of SmB/B′ and Cdt1. (D‐F) After the thymidine treatment, SmB/B′ generally matches that of nuclear Geminin expression, although, SmB/B′‐positive cells with unclear nuclear localization of Geminin are also seen (arrow). In (E), cells with apparent chromatin condensation judged by DAPI signals are indicated by arrowheads, in which both Geminin and SmB/B′ are strongly positive in the nucleus. In (F), cells at anaphase to telophase with aligned chromatins are prominent, in which SmB/B′ and Geminin are positive in the whole cell. (G and H) After the 2,3‐DCPE treatment, Geminin is mostly in the perinuclear region and SmB/B′ is only weakly stained (G). Arrow in (G) indicates a cell expressing SmB/B′ in the cytoplasm. Cdt1 is co‐expressed with SmB/B′ (H). Scale bars, (A and D‐F) 50 μm; (B, C, G, H) 20 μm

We further addressed the mixed pattern of SmB/B′ expression in relation to the cell cycle using cell cycle regulators Geminin and Cdt1 as markers. Geminin is localized to the cytoplasm in the G1 phase before the DNA synthesis, then translocated to the nucleus during the S/G2/M phases to prevent further DNA replication, after which it is degraded.^38^, ^39^ Cdt1 accumulates in the nucleus during the G1 phase and allows DNA synthesis to begin, then degrades to prevent further DNA replication in the S phase.^40^, ^41^ In the control condition of HeLa cells without cell cycle synchronization, Geminin was expressed either in the nucleus or faintly in the perinuclear region, reflecting the S/G2/M or G1 phase, respectively (Figure 9A,B).^38^ Cells with Geminin in the nucleus were SmB/B′‐positive, whereas those with Geminin in the perinuclear region were generally SmB/B′‐negative (Figure 9B), suggesting a possible correlation of SmB/B′ expression to the cell cycle. Although, in some SmB/B′‐positive cells, the nuclear localization of Geminin was not very clear (Figure 9A,D), probably due to the gradual translocation of Geminin during the transition from the G1 to S phase. The expression of Cdt1 largely correlated to that of SmB/B′ (Figure 9C). To synchronize the cell cycle, cells were treated with Thymidine to induce G1 arrest^42^ and, after the withdrawal of Thymidine, they were cultured for further 2, 4, or 8 h to allow cell cycle progression. Geminin‐positive nuclei became apparent over time with prominent mitotic cells at 8 h, which was accompanied by strong SmB/B′ expression (Figure 9D‐F). In another cell cycle synchronization, cells were treated with 2[[3‐(2,3‐dichlorophenoxy)propyl]amino]ethanol (2,3‐DCPE), which causes S phase arrest.^43^ 2,3‐DCPE treatment resulted in Geminin expression excluded from the nucleus in most cells, as anticipated, indicating the cells were at the end of the G1 phase or early S phase. In such cells, the nuclear SmB/B′ expression was weak, and some cells showed SmB/B′ staining in the cytoplasm (Figure 9G). The cells that express Cdt1, which is indicative of the late G1 to early S phase, expressed SmB/B′ in the nucleus (Figure 9H). These results suggest that SmB/B′ is strongly expressed in the nucleus at the late G1 to G2 phases and dispersed in the cell during the M phase when the nuclear membrane breaks down.^21^, ^22^, ^23^ The weak expression in the nucleus is likely at the G1 phase when Geminin is not in the nucleus.

**FIGURE 10:**
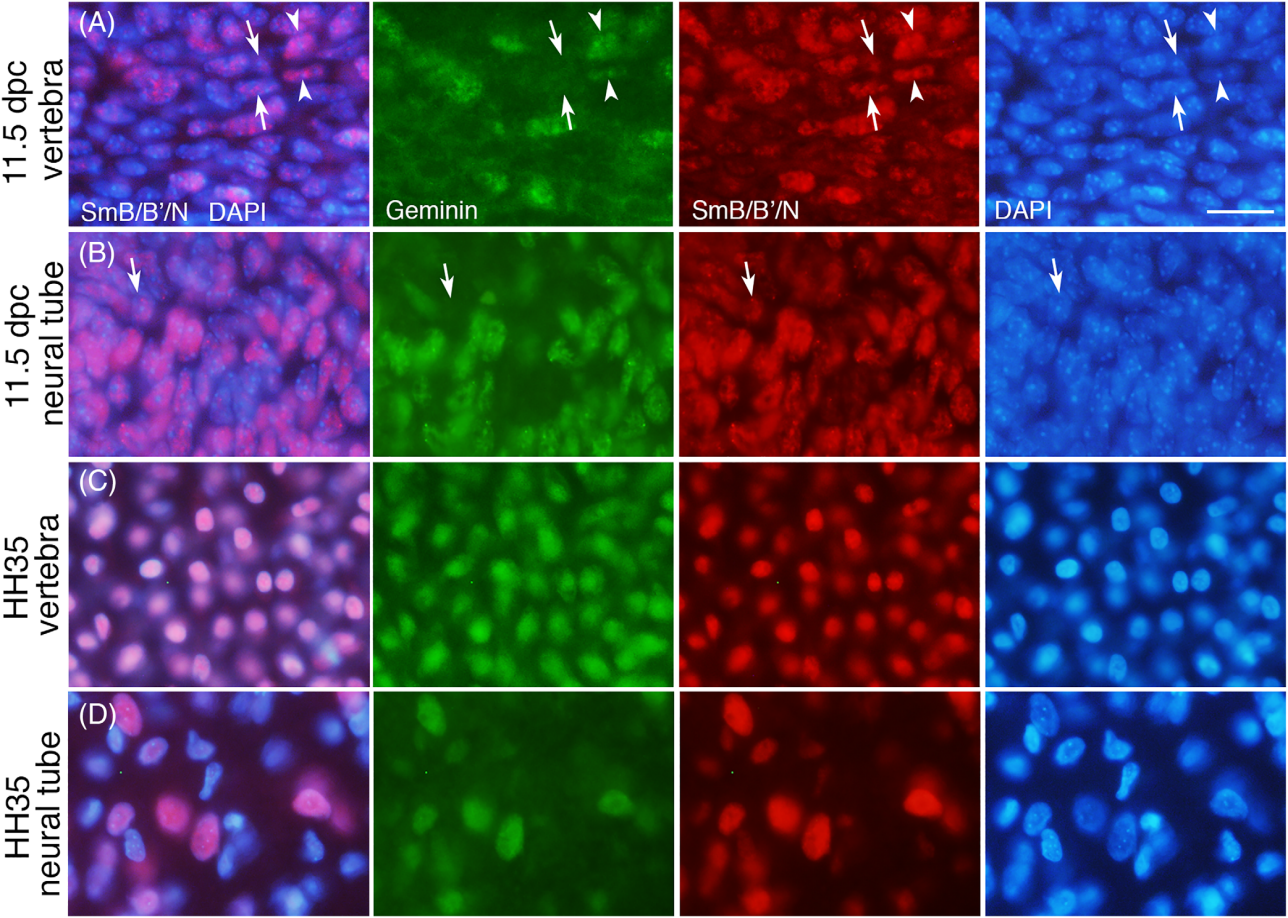
Expression of SmB/B′ and Geminin in the vertebra and neural tube. Transverse sections of the mouse (A and B) and chick (C and D) vertebral cartilage and ventral neural tube at the indicated stage, stained with anti‐SmB/B′ antibodies A5 (red), Geminin (green) and DAPI (blue). The staining of SmB/B′ and Geminin generally overlap (arrowheads in A for example), except for some cells that are SmB/B′‐positive and Geminin‐negative or faint staining (arrows in A and B). Scale bar, 20 μm for all panels

The co‐relation of SmB/B′ and cell cycle was examined in vivo in the mouse and chick neural tube and vertebral cartilage where we observed sporadic expression patterns (Figure 10). The trend of co‐expression of SmB/B′ and Geminin was largely the case, although, Geminin‐negative (indicative of the G1 phase) and SmB/B′‐positive cells were also noted (Figure 10) similarly to the in vitro result (Figure 9). It is, thus, suggested that SmB/B′ is expressed strongly in S/G2/M phases and maybe, not always though, lowered at the G1 phase.

## 3 DISCUSSION

Despite the omnipresent requirement of the spliceosome in all cells in the body, our immunohistochemical study has revealed that SmB/B′, one of the core spliceosomal proteins, is not necessarily expressed ubiquitously in embryos; rather, in a tissue‐ and cell‐type‐specific manner with various degrees of strengths. In early embryogenesis after gastrulation, the expression appeared ubiquitous and homogeneous. As the embryos develop and cells differentiate, a variety of patterns emerged. Sporadic expression was observed as a mix of strong, weak and apparently negative nuclei in developing cartilages and the ventral column of the neural tube (Figures 1 and 2). One suspected mechanism for this is related to cell cycle arrest, as both neurons and chondrocytes may exit the cell cycle during development. In vitro analysis on the relation between SmB/B′ and cell cycle in this study and others also suggested some correlation. It has been reported that *SNRPB* knockdown affects cell cycle progression and causes cell cycle arrest in a severe depletion.^44^ It is, however, yet to investigate whether a spontaneous cease of SmB/B′ expression occurs in vivo or not, given that post‐mitotic cells would require splicing. Our immunohistochemical studies showed that the nuclear localization appeared not very strict in tissues such as the liver and lungs (Figure 3). Why subcellular localization differs between cell types is also yet to be studied.

The expression of *SNRPB* at the transcriptional level varies in normal adult organs and cell types.^11^ Our study confirmed the diverse expression within embryos at the protein level and further demonstrated different subcellular patterns among various tissues and organs.

### 3.1 The relation to the CCMS phenotype

A high level of SmB/B′ in specific tissues may reflect a heavy reliance on normal functionality and hence confer susceptibility of the tissue to reduced expression in CCMS patients. In this scenario, the phenotype would be presented in structures with high expression levels of SmB/B′. Alternatively, tissues with higher levels of expression may be tolerant to the reduced level of expression, rendering regions at lower expression levels to be more vulnerable to *SNRPB* mutation. This study showed that the regions affected by CCMS; rib cartilages, vertebrae and the mandible, as well as the mesenchymal cells in the palate, showed a clear nuclear‐specific and strong yet sporadic pattern. However, such an expression pattern does not correlate to the CCMS phenotype; for example, the cells in the neural tube also showed a nuclear‐specific and sporadic pattern but neural deficits are not reported as part of the CCMS phenotype. In addition, cartilages of the primordial scapula and limbs also showed a sporadic pattern and yet they are not affected in CCMS. Hence, this study did not detect a clear correlation between the phenotypic regions in CCMS and the expression level and patterns of SmB/B′. A more extensive stage‐specific investigation is needed. Nonetheless, the present study highlighted the unique sporadic expression of SmB/B′ in developing cartilages and palatal mesenchyme. Concerning reliance vs. tolerance, the high demand for splicing has been suggested to correlate more closely with spliceosomal syndromes. For example, the retina, a tissue often affected by spliceosomal mutations,^45^ exhibits a high level of splicing activity.^46^ Another example supporting the theory that the high demand for the spliceosome is causative of disease is the affected snRNP assembly during the development of the CNS. Spinal muscular atrophy is caused by mutations of *survival motor neuron* (SMN) which is normally required for the metabolism of snRNPs and expressed at the highest levels at the most crucial stage of development for the phenotype presentation.^47^ In this study, the CCMS phenotype‐presenting structures such as developing cartilages and palatal mesenchyme were found to exhibit high levels of SmB/B′ expression in the nucleus although not homogeneously in all cells. This may confer a high demand and susceptibility because some cells show reduced levels of expression even in the normal course of development, as such, further reduction by mutations may be detrimental.

### 3.2 Cell cycle

Given the possible relation of SmB/B′ expression to the cell cycle, it is plausible that the impact of reduced SmB/B′ expression is related to the cell cycle length. Embryos' cell cycles are generally fast, especially at the early stages of embryogenesis.^48^ On the other hand, many adult cells and immotile cell lines in vitro are relatively slow in the cell cycle; some adult cells are post‐mitotic. Even in embryos, some cells such as differentiating cartilages and neurons are post‐mitotic. Cells with slow cell cycles (with the long G1 phase) or post‐mitotic (at G0) cells would have a longer duration of the SmB/B′‐negative condition, hence, they may be more susceptible to the reduced SmB/B′ expression and have a stronger impact on cells. Hypertrophic chondrocytes in embryos may be affected more strongly than other cell types in *SNRPB*‐mutated individuals. In cells with a high speed of cell cycle like those in young embryos, the G1 phase is likely short, thereby the shortage of SmB/B′ may not be deteriorating. The homogeneous and strong expression of SmB/B′ in the nucleus at the young stages of embryos (Figure 1) agrees with this. If reduced SmB/B′ expression was crucial in such fast‐proliferating cells, *SNRPB*‐mutated embryos would not survive through the primary morphogenesis such as gastrulation and axis elongation in humans (corresponding to embryonic Day 1‐2 in chick embryos). However, it is puzzling how cells in adult CCMS patients can cope with the shortage of SmB/B′ given that most adult cells are at the G0 phase or long G1 phase. Another question is why CCMS phenotypes are only congenital, and the patients do not develop further clinical problems progressively after birth. There must be other elements of factors responsible for the phenotype presentation by *SNRPB* mutations in CCMS as well as for normal SmB/B′ function.

## 4 EXPERIMENTAL PROCEDURES

### 4.1 Immunohistochemistry

Fertilized chick eggs (brown) were incubated for 2‐10 days and embryos were staged according to Hamburger and Hamilton,^27^ whereas mouse embryos were harvested at 11.5 and 13.5 dpc (Theiler stage TS19 and TS21, respectively)^49^ from the CD‐1 strain, both following the regulation of University of Bristol. Embryos were fixed with 4% paraformaldehyde in PBS overnight. Primary antibodies used in this study were; SmB/B′ antibodies A5 (Santa Cruz sc‐374 009), 12F5 (Santa Cruz sc‐130 670), and Y12 (Invitrogen MA5‐13449). For immunohistochemistry on embryo sections, A5 antibodies were used on most sections, except that Y12 was used for Figure 3K. To note, anti‐SmB/B′ antibodies may cross‐react with SmN which is encoded by *SNRPN* in mice and homologous to SmB'. Chickens do not express *SNRPN*/SmN. At least three embryos were collected from each stage and more than five histological sections were analyzed for each stage. Immunohistochemical staining was performed on cryosections.

### 4.2 RNA in situ hybridization

In situ hybridization was performed either on whole mounts or cryosections, following an established protocol.^50^, ^51^ Antisense and sense RNA probes for *SNRPB* were synthesized using a chick EST clone ChEST385l19 which contains 30 bp of 5′UTR, 723 bp of the full‐coding sequence and 140 bp of 3′UTR. ChEST287o3 clone was used to make an RNA probe for *SNRNP200*. These clones were obtained from Source Bioscience (UK).^52^

### 4.3 Western blotting

For Western blotting, chick embryos at HH10‐13 (E2), HH27 (E5), and HH31 (E7) were dissected and homogenized in RIPA buffer (150 mM NaCl, 25 mM Tris‐HCl, pH 7.6, 1% NP40, 1% Sodium deoxycholate, 0.1% SDS, proteinase inhibitor [Sigma P8340]), after which the extraction was span at 15 000 *g* and a part of the supernatant was used for total protein quantification (Pierce 23225). The protein concentration of each sample was adjusted to the same level using RIPA buffer, after which the denaturing sample buffer was added and subjected to Western blotting. ImageJ software was used to quantify the intensity of bands.

### 4.4 In vitro cell culture

HEK293, Saos‐2 and HeLa cells, all of human origin, were obtained from ATCC and cultured in DMEM (Merck) with 10% fetal calf serum. For double‐thymidine treatment, cells were incubated with 2 mM thymidine for 18 h, then after 8 h of an interval in the fresh medium, thymidine was again added for another 20 h^42^ before the subsequent incubation. For treatment with 2,3‐DCPE,^43^ cells were incubated for 30 h with a complete medium containing 2,3‐DCPE at 20 μM. Anti‐Geminin antibody (Abcam ab195047) and anti‐Cdt1 antibody (Abcam ab202067) were used.

## AUTHOR CONTRIBUTIONS

**Benedict R. H. Turner:** Formal analysis (lead); investigation (lead). **Charlotte Mellor:** Formal analysis (equal); investigation (equal). **Clara McElroy:** Formal analysis (equal); investigation (equal). **Natalie Bowen:** Formal analysis (equal); investigation (equal). **Wenjia Gu:** Formal analysis (equal); investigation (equal). **Chris Knill:** Formal analysis (equal); investigation (equal). **Nobue Itasaki:** Conceptualization (lead); data curation (lead); formal analysis (equal); funding acquisition (lead); investigation (lead); methodology (lead); project administration (lead); resources (lead); supervision (lead); validation (lead); visualization (lead); writing – original draft (lead); writing – review and editing (lead).
